# TRIPS, the Doha declaration and paragraph 6 decision: what are the remaining steps for protecting access to medicines?

**DOI:** 10.1186/1744-8603-3-3

**Published:** 2007-05-24

**Authors:** Vanessa Bradford Kerry, Kelley Lee

**Affiliations:** 1 Harvard Medical School, 260 Longwood Avenue, Boston MA 02115, USA; 2 Centre on Global Change and Health, London School of Hygiene & Tropical Medicine, Keppel Street, London, WC1E 7HT, UK

## Abstract

**Background:**

The World Trade Organisation's Declaration on the TRIPS Agreement and Public Health (known as the Doha Declaration) of 2001, and subsequent Decision on the Interpretation of Paragraph 6 reached in 2003, affirmed the flexibilities available under the Agreement on Trade Related Property Rights (TRIPS) to member states seeking to protect public health. Despite these important clarifications, the actual implementation of these measures to improve access to medicines remains uncertain. There are also concerns that so-called TRIPS-plus measures within many regional and bilateral trade agreements are further undermining the capacity of the poor to access affordable medicines.

**Methods:**

The paper reviews policy debates among governments, nongovernmental organisations and international organisations from 1995, and notably since 2003, surrounding access to medicines and trade agreements. The provisions for protecting public health provided by the Doha Declaration and Paragraph 6 Decision are reviewed in terms of challenges for implementation, along with measures to protect intellectual property rights (IPRs) under selected regional and bilateral trade agreements.

**Results:**

While provisions, in principle, were affirmed for member states under the TRIPS agreement to protect public health, numerous challenges remain. Implementation of the flexibilities has been hindered by lack of capacity in many LMICs. More intransigent have been stark inequalities in power and influence among trading nations, leaving LMICs vulnerable to pressures to permit the globalization of IPRs in order to protect broader trade and economic interests. Such inequalities are apparent in proposals or adopted TRIPS-plus measures which re-establish the primacy of trade over public health goals.

**Conclusion:**

Despite being hailed as a "watershed in international trade", the Doha Declaration and Paragraph 6 decision have not resolved the problem of access to affordable medicines. The way forward must begin with a simplification of their content, to enable actual implementation. More fundamentally, once agreed, public health protections under TRIPS must be recognised as taking precedent over measures subsequently adopted under other trade agreements. This requires, above all, setting aside such protections as a basic need and shared goal from trade negotiations at all levels.

## Background

The issue of access to medicines remains at a crossroad between the ongoing globalisation of intellectual property rights (IPRs), and significant demand for drugs to meet critical public health needs among the world's poor. Campaigning by the governments of many low and middle-income countries (LMICs), alongside nongovernmental organisations (NGOs), has centred on the potential for trade agreements, notably the Agreement on Trade-Related Aspects of Intellectual Property Rights (TRIPS), to hinder the availability of affordable medicines. In 2001 the Declaration on the TRIPS Agreement and Public Health (known as the Doha Declaration), affirmed the right of member states of the World Trade Organisation (WTO) to interpret and implement TRIPS in a manner supporting the protection of public health and, in particular, access to medicines [1]. While initially well-received, consternation soon arose over interpretation of a specific paragraph of the Doha Declaration on compulsory licensing [2]. After two years of further deliberation, the WTO Decision on the Interpretation of Paragraph 6 was announced in 2003 specifying when countries can import drugs produced elsewhere under compulsory licensing [3]. The WTO describes the Paragraph 6 decision as removing the "final patent obstacle to cheap drug imports."[4]

With one-third of the world's population still lacking access to essential medicines, a figure rising to over 50% in Asia and Africa, [5] for the public health community the problems are two-fold. The first is the capacity of LMICs to actually implement the flexibilities afforded under TRIPs, the Doha Declaration and Paragraph 6 decision amid stark inequalities in health resources and the world trading system as a whole. These include provisions for compulsory licensing, parallel importing and addressing imbalances in research and development (R&D). The pending ratification of the Paragraph 6 decision, from an interim solution to permanent amendment, is accompanied by much uncertainty – will the protections be accessible under the currently proposed system? The second are concerns about the undermining of the above hard won flexibilities by provisions being adopted under various bilateral and regional trade agreements. Known as "TRIPS plus" or "WTO plus" measures, the standard of IPRs being negotiated and even adopted under other trade agreements are more restrictive of public health protections. These two sources of concern have led to increased, rather than lessening, tensions between the public health and trade policy communities.

This paper begins by briefly reviewing progress to date on the public health protections available under the TRIPS agreement. It describes how, despite these important clarifications, there remain concerns about the capacity of LMICs to implement specific measures. The paper then considers the further threat posed by TRIPS-plus measures and calls for their critical assessment. Central to debates about implementation and TRIPS-plus is an understanding of fundamental imbalances in power and influence, both within and across countries, defining what interests can and cannot influence trade policy decisions. The paper concludes by reviewing potential ways forward to ensure that access to medicines by the poor is secured within all trade agreements.

### TRIPS, the Doha Declaration and Paragraph 6 decision: When public health protection takes primacy over trade

The TRIPS agreement came into effect in January 1995, alongside the creation of the WTO, to facilitate trade through the creation of a comprehensive multilateral agreement on IPRs including patents, trademarks and copyright. Prior to its implementation, IPR protection was unevenly recognised in many countries. On patents, TRIPS extended minimum standards of protection for any inventions, whether products or processes, in all fields of technology without discrimination, subject to the normal tests of novelty, inventiveness and industrial applicability [6]. This includes the requirement by all WTO members to make patents available for pharmaceutical innovations. Alongside the conferment of patent rights for a period of twenty years from the filing date, TRIPS establishes procedures and remedies for patent holders to enforce their rights.

In principle, TRIPS is intended to create a "level playing field" of mutually recognised IPRs among all member states, encouraging trade and, in turn, economic growth. From its inception, however, the agreement has been the subject of intense controversy, focused on how its provisions affect the ability of the world's poor to access affordable medicines [7,8]. Before 1995, LMICs engaged in a robust trade in generic and recently marketed drugs produced in countries where patent rights were unrecognised. For the importing country, this trade was a source of cheaper medicines, especially critical to countries under severe resource constraints facing major public health problems such as HIV/AIDS. Compliance with TRIPS since 1995 has required WTO member states to restrict such trade, and to grant to patent holders exclusive rights to produce and sell protected drugs [9,10]. For public health proponents, TRIPS enhanced the interests of transnational pharmaceutical companies and industrialised countries with large pharmaceutical industries, notably the US, Japan and European Union, at the expense of access to affordable medicines by millions in genuine need.

Attempts to settle the concerns around public health protections led to the Doha Declaration in 2001, followed by the Implementation of the Paragraph 6 Decision in 2003. Combined, the two declarations provide clarifications on the need for, and provisions available, to access generic medicines. One of the most important results was a waiver of Article 31(f) of the TRIPS agreement which states that a compulsory license can only be issued for primarily domestic use. This paragraph precluded generic drug production for export to countries without their own domestic capabilities, leaving the poorest countries without access to generic medicines. The waiver allowed a country to issue a compulsory license for either domestic use or export, on the basis of public health need [11].

The Doha Declaration and Paragraph 6 decision were initially hailed as a triumph by public health advocates [12]. The agreements appeared to distinguish drugs from other traded commodities, and to secure the right of WTO member states to uphold flexibilities contained within the TRIPS agreement for the purpose of protecting public health. Evidence of the positive impact of the agreements was a decline in complaints against countries for inadequate IPR protection, registered by the US Trade Representative, from five in 1999 to one in 2002 [13].

### Implementing the Doha Declaration in a world of inequality

Despite the affirmations provided by the Doha Declaration and Paragraph 6 decision, there remain a number of difficulties for LMICs seeking to implement them in practice.

#### Compliance with TRIPS by LMICs and least developed countries

While the Doha Declaration extends the transition period for compliance with the TRIPS agreement by least developed countries (LDCs) to 2016, it does *not *affect the original timeline of 2005 for compliance by other LMICs. The distinction between LDCs and other LMICs can be misleading. LDCs is a designation created by the United Nations to determine which countries are in greatest need of aid. The list of around fifty countries is reviewed every three years by the Economic and Social Council (Ecosoc) according to criteria such as low income, weak human resources and low level of economic diversification [14]. However, many LMICs such as Kenya and Nigeria not officially classified as LDCs remain very poor, and aggregate national data obscures health needs among poor populations within them.

The 2005 date of compliance for most LMICs includes countries that are major suppliers of generic drugs such as India, Brazil and China. India is the fourth largest producer of prescription drugs by volume, supplying 22% of the world's generics and a significant proportion of vaccines to the developing world [15]. Major producers in India include Ranbaxy with US$1.2 billion sales in 2005, 76% earned from overseas markets [16,17]. China had over 4000 pharmaceutical factories in 2003, and is a world leader in producing active pharmaceutical ingredients (API) for first line ARVs, as well as producing many second line ARVs [15]. Brazil's generic industry, comprised of 37 national and 12 foreign companies, is also rapidly growing, spurred by both domestic demand and export potential [18]. Since 2005, unauthorised production and sale of generic versions of drugs under patent by most LMICs has not been permitted. While most drugs on WHO's essential drug list were patented before 1995, and therefore unaffected by the new measures, stronger IPR protection affect the patent status of new and future drugs. Restricting the production of generic drugs in compliance with TRIPS also reduces competition, again increasing prices and reducing affordability [19]. The cost of ARVs are the most frequently cited, with generic versions of second line treatments costing as little as US$140 per year (compared with US$30 000 for patented versions) [20]. However, a range of other treatments are facing higher prices. For instance, generic versions of the drug Gleevac^® ^(iminatib mesylate), a life-saving treatment for chronic myeloid leukaemia, has reduced the price from US$2000 to US$200 per month [21]. A legal challenge by Novartis, of the Indian Patent Office's denial of a patent for the drug, given India's compliance with TRIPS in 2005, brought protests by patient advocacy groups and NGOs [22].

#### Compulsory licensing and parallel importing

While the Doha Declaration clarifies the right of LMICs to engage in compulsory licensing and parallel importing, there remains much trepidation about its use in practice. Countries reliant on trade with powerful trading partners have remained reluctant to exercise available flexibilities for fear of incurring their wrath in other trade areas. Brazil's efforts to freely provide ARVs are an often cited example of how the declaration has strengthened the position of LMICs. The Brazilian policy, announced in 1996, was made possible by the production and import of generic first-line and second-line treatments [23]. With Brazilian compliance to TRIPS in 2005, the latter was no longer permitted and the cost of second-line became problematic. Threatening to introduce compulsory licensing, as permitted under the Doha Declaration, the Brazilian government pressured Abbott, Merck and Roche (manufacturers of lopinavir, indinavir, nelfinavir and saquinavir respectively) to substantially reduce prices, thus enabling more than 100,000 people to receive free treatment [24]. In this case, while the threat of compulsory licensing yielded concessions by pharmaceutical companies, the flexibilities remained untested in practice.

It was not until 2005 that the first country issued a compulsory license under the waiver on the grounds of protecting public health. As countries have scrambled to stockpile the anti-viral drug Tamiflu^® ^(oseltamivir), amid fears of a potential influenza pandemic, international pressure was exerted on the patent holder Roche to issue voluntary licenses to permit manufacture by other companies. As a country potentially among those most immediately affected by a pandemic, Taiwan decided to use the flexibilities affirmed by the Doha Declaration to secure access to Tamiflu^®^. Yet in doing so, the government conceded provisos which suggested caution on its part. Despite a clear public health rationale for the action, the Taiwanese government remained concerned to minimise potential damage to its image as a trading economy. For example, manufacture would be for domestic purposes only, limited to the end of 2007 and subject to "appropriate" license fees to Roche. Moreover, Taiwan would use up all Roche supplied drugs before using locally produced supplies, and the compulsory license could be revoked once agreement on a voluntary license was reached. As stated by the Tipo deputy director-general, "Under these circumstances, the Department of Health will not need to use Tamiflu^® ^from sources other than Roche unless a pandemic hits early next year and stockpiles are used up quickly." [25]

The most notable action to date, to assert the waiver of Article 31(f), has been by the Thai government which authorised the Government Pharmaceutical Organisation in November 2006 to manufacture generic versions of efavirenz (Stocrin^®^) until 2011, and to import generic versions from India until domestic production comes into line [26,27]. While the manufacturer Merck conceded that the action was in compliance with TRIPS, the company claimed the government did not engage in sufficient consultation to allow, for example, negotiation on a possible reduced price for the drug. The US government also questioned the validity of the license, and pressed Thailand to rescind the decision and negotiate with Merck. Unwavering, Thailand took a step further in January 2007, issuing two further compulsory licenses for Kaletra^®^, patented by Abbott, and Plavix^® ^patented by Sanofi-Aventis. These actions are seen as the most serious attempt to date to override patents [28]. Abbott initially responded by withholding a number of new medications from the Thai market including the heat stable form of Kaletra^®^. The company has since offered the medicine to Thailand and 39 other countries for US$1000 per patient per year, although it continues to withhold other medications.

The experiences to date suggest that there remains general reluctance among LMICs to fully test the flexibilities for compulsory licensing available under TRIPS. In an increasingly global economy, maintaining one's standing as a trading partner committed to IPR protection has so far taken precedence over access to medicines. The strong reaction to the Thai government's action by the US and transnational pharmaceutical industry reflects the degree of pressure on countries to resist the use of TRIPS flexibilities.

#### Data exclusivity and regulatory approval

Data exclusivity refers to the keeping confidential by drug regulatory authorities of data on the safety and efficacy of a new medicine for a set period. This data would be especially useful to generic producers which need only demonstrate through such data that their product is therapeutically equivalent to the original (bio-equivalency). Without access to registered data, generic producers must either wait the given time period or replicate the studies themselves. In principle, the market power of data exclusivity is less restrictive than patents since it does not prevent companies from creating their own data. In practice, access to such data substantially reduces time, expense, and effort needed for registering new drugs [29]. Increasing the requirements around data exclusivity, in short, effectively provides the data holder monopoly status during which time it can market its product without competition from generic products.

#### The extension of patent rights

Under Article 33 of TRIPS, "the term of protection shall not end before the expiration of a period of twenty years" from the filing date. This is the period during which the product can be marketed with exclusive patent rights. However, the length of the protection period can be a reduced by two administrative procedures – the patent examination process and marketing approval process. To avoid "unwarranted curtailment" of the protection period, the TRIPS agreement states that a patent should be granted within a "reasonable period of time" (Article 62(2)). Prior to compliance with TRIPS, unwarranted curtailment was not an issue given that US law granted the period of protection from the date the patent is granted. Following compliance with TRIPS, the US Patent Term Guarantee Act was adopted in 1999 which permits the protection period to be extended if a patent is not granted within three years.

#### The research and development gap

Neither the Doha Declaration nor Paragraph 6 decision address the fundamental issue of underinvestment in R&D for health conditions that predominantly impact LMICs. Between 1975 and 1997, 1,223 new chemicals were launched on the market. Of the 31% which were therapeutic innovations, only 1% was helpful for tropical diseases [30]. R&D remains heavily concentrated in a small number of large pharmaceutical companies located in high-income countries seeking to serve those markets [31]. For example, there are more drugs in the pipeline for brain tumours than for tuberculosis which is one of top killers globally and especially in the developing world [32,33]. As profit-making commercial concerns, these companies focus on markets which promise the greatest economic return. Currently, 90% of research funds go to only 10% of the world's disease burden [49]. For most LMICs, lack of domestic R&D capacity and purchasing power means a lack of drug development to meet significant health needs.

#### Need for National Laws

The lack of appropriate legislation in many LMICs to enshrine the protections under the TRIPS agreement, Doha Declaration and IDDT remains a key challenge. National legislation is essential because many provisions are permitted only if written into law. Currently, many LDCs have stricter IPR protection than is minimally required by TRIPS [20]. Of thirty African LDCs, only two do not grant patents for pharmaceuticals [34]. Furthermore, LMICs can only assert available flexibilities and enhance their purchasing power if appropriate national drug policies are in place, backed by a legislative framework concerning such issues as use of generics, drug pricing and taxation.

In this context, the key priorities for strengthening national legislation in LMICs should include provisions for compulsory licensing for both import and export, definition of international exhaustion of rights and parallel importing, early working policies and, for LDCs, how to best use the available transitional period for compliance. The option to use compulsory licensing, in particular, is being hindered by complex legal and administrative barriers including a failure to write compulsory licensing into law. For example, Panama has no national legislative provision for issuing a compulsory license, while Honduras does not include compulsory licensing as a remedy for anti-competitive practices or unfair competition [35]. To remedy this, countries must outline strong government provisions with comprehensive and full entitlements provided under TRIPS, including authorization for patents for public, non-commercial use and fast-track authorization without long negotiations [36]. This requires clear and straightforward procedures that do not suspend execution of a compulsory license if appealed against. This would include writing into legislation the onus for proof of patent infringement on the patent holder [37]. Equally critical is for countries *with *manufacturing capabilities, which have been compliant with TRIPS since 2005, to establish legislative and administrative frameworks for allowing compulsory licensing for export purposes. These countries include India, China, Brazil, Canada, South Africa and Singapore [38,23].

### Divide and conquer: The undermining of public health protections through bilateral and regional trade agreements

Along with the above barriers to implementing TRIPS flexibilities, there is substantial concern among public health advocates about the spread of so-called "TRIPS-plus" measures. As efforts to progress trade liberalisation through multilateral channels have stalled since 2003, major industrialised countries have pursued negotiations for bilateral and regional trade agreements outside of the WTO. Seeking to fuel economic growth through trade, governments of LMICs have agreed to such measures in exchange for access to potentially lucrative export markets for key sectors such as agriculture and textiles. For the public health community, however, provisions to protect access to medicines have been bargained away in a number of ways.

First, the scope for compulsory licensing and parallel importing has been a particular focus of TRIPS-plus restrictions, narrowing the circumstances when parties are permitted to use these measures. Under negotiations for a Free Trade Agreement of the Americas (FTAA), for example, it is proposed that compulsory licensing would only be permitted when the patent on a product has expired or in situations of "national emergency", with a body to be set up over and above the WTO to rule on disputes [39]. Grounds permitted under agreements between the US and Australia, Jordan and Singapore are limited to anticompetitive practices, public non-commercial use, national emergency or other circumstances of extreme urgency [40]. Under the US-Australia FTA, drugs produced under compulsory license in Australia are excluded from parallel importation, even to alleviate a public health crisis in a neighbouring country [41,42]. Similar measures have been agreed between the US and Morocco, and US and Singapore [43], and are being discussed in US negotiations with dozens of additional countries. The same concerns arise under trade agreements negotiated by EFTA [44] and the European Union with the Southern African Customs Union (SACU), Chile, Morocco, Mexico, the Palestinian Authority and Jordan [45,46].

Second, TRIPS-plus measures increase provisions concerning data exclusivity, enabling large pharmaceutical companies to prevent or delay generic competition. While TRIPS already provides for protection of such data, many bilateral and regional agreements extend both the scope and length of such protections. For example, the US-Australia FTA includes a five-year protection period for "undisclosed" pharmaceutical test data. The period among EU member states is even longer at eight to ten years [47]. Other restrictions negotiated include extending the protection of data disclosed through the grant marketing procedures (versus data undisclosed covered by TRIPS), extending data protection past patent expiry to offset time lost during marketing approval (US-Chile, US-Jordan, Central American FTA), and/or prohibiting reliance on prior test data of both patented and off-patent products by market approval authorities. These stronger protections raise concerns because they reduce the capacity of a country to issue or use compulsory licensing for unpatented drugs. If required to await expiry of data exclusivity, a country in effect is unable to make effective use of a license [47]. According to Médicins sans Frontièrs, for example, in Guatemala, generic manufacturers for most ARVs need to wait fifteen years from the date of approval of the original medicine in the country before obtaining registration of their own version of the medicine. In Jordan, an analysis of 103 medicines registered and launched since the signing of the US-Jordan FTA in 2001, found at least 79% have no generic competition as a consequence of data exclusivity introduced under the agreement [48].

A related issue is that many bilateral and regional trade agreements do not allow the so-called Bolar Provision. This provision, also known as "early working", permits the use of a patent protected invention or process and/or data without authorization in order to facilitate regulatory approval of a generic product *before *the patent expires. This allows a generic product to enter the market more quickly, speeding access to cheaper drugs. Under TRIPS-plus measures, a patent owner must consent to marketing approval for a generic version during the patent term.

Third, the period of patent protection has been extended under TRIPS-plus measures. Bilateral agreements between the US and Jordan, Chile, Australia, and proposals under the FTAA, all effectively extend the period of patent protection [49]. A related form of patent extension is "evergreening," a term which refers to patent protection of *inventions*, as opposed to *medicines *which may in fact have multiple patents. "New use" for existing compounds, or a change in the dose or form, can be the basis for applying for an extension of the patent protection period, thus preventing generic versions of the drug from being produced. While not permitted under TRIPS, many FTAs include the "new use" clauses. Even if an application for "new use" does not succeed, the process of application can create considerable delays, especially when applications become embroiled in disputes over a potential patent violation [46].

The available flexibilities under the TRIPS agreement to protect public health, in short, face erosion by the negotiation and agreement of TRIPS-plus measures. Major industrialised countries, seeking to protect the interests of transnational pharmaceutical companies, have pursued a "divide and conquer" strategy. There is need to consider how the public health community must act to prevent the goal of access to medicines from being further undermined.

### Reaffirming access to medicines as a global priority: What can be done?

The limited progress in improving access to medicines through TRIPS, as affirmed by the Doha Declaration and Paragraph 6 decision, points to the need for reassessment. In February 2004, the WHO Director-General established the Commission on Intellectual Property Rights, Innovation and Public Health to review the available evidence and recommend ways forward to improve systems for developing and accessing drugs in LMICs. The Commission considered access to medicines within a broader context of industry structure and market incentives, recognising that IPRs are only one means of stimulating action. In its final report, the Commission made sixty recommendations organised into five categories: (a) the *discovery *of new health-care products; (b) the *development *of drugs from pre-clinical and clinical research, and the regulatory process; (c) the *delivery *of new and existing products to LMICs; (d) the *fostering of innovation *in the developing world; and (e) the *roles and responsibilities of WHO *in leading ways forward.

**Figure 1:**
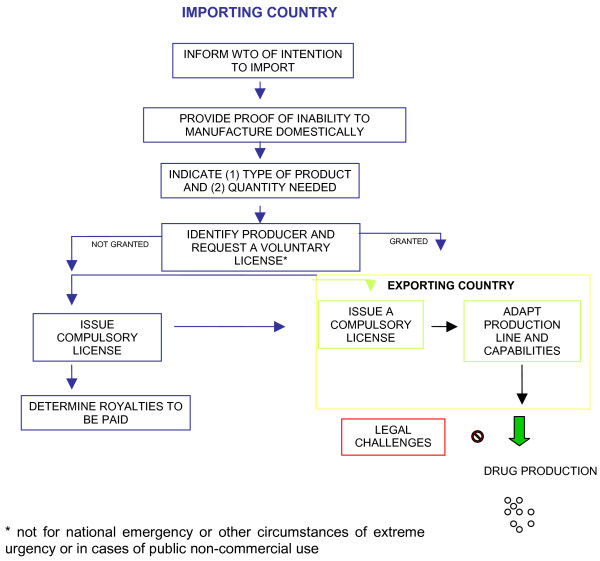
Requirements under tabled amendment to Paragraph 31(f) of TRIPS.

While it is beyond the scope of this paper to provide a detailed assessment of all recommendations, options for implementing the Doha Declaration and Paragraph 6 decision, and the threats posed by bilateral and regional trade agreements, need to be considered alongside them. The limitations of the two agreements have become apparent in efforts to adopt them as a permanent amendment to Article 31(f) of the TRIPS agreement. Negotiations on the amendment began in early 2004, with initial hopes that they would be concluded within six months. While the discussions have not formed part of the Doha Development Round of multilateral trade negotiations, the issue soon became subsumed as a bargaining chip, in the run up to the Hong Kong Ministerial Conference of 2005, by powerful trading countries seeking concessions on other issues, notably agricultural subsidies,. Efforts to find a "permanent solution" to compulsory licensing at the WTO TRIPS Council in October 2005 stumbled on the realisation by a growing number of LMICs that the tabled amendment was overly cumbersome (see Figure 1). According to Médicins sans Frontières (MSF), they placed a "burden on drug procurement [that] could discourage rather than encourage generic production." [50] For example, if a country wanted to issue a compulsory license for efavirenz, tenofovir and lamivudine as a triple combination therapy for HIV/AIDS, this would require separate applications for each medicine involving three different manufacturers (Bristol-Myers Squibb, Gilead Sciences and GlaxoSmithKline). Moreover, as the procedure is required on a drug-by-drug and country-by-country basis, any economies of scale would be lost, potentially increasing prices and decreasing incentives to exporters. African countries, supported by other LMICs including Brazil and India, unsuccessfully tabled a proposed amendment to Article 31(f) that excluded such burdensome requirements. They also sought to exclude provisions set out in a statement by the Chair of the General Council, Carlos Pérez del Castillo, made on 30 August 2003 alongside the Paragraph 6 decision [51]. The US, however, strongly opposed the removal of the Chair's statement, considering it an integral part of the decision itself. Efforts by EU member states to informally table a "middle ground" approach failed to break this impasse and, after further pressure on LMICs, the original amendment was put forward for ratification. With a deadline of December 2007, to date only three countries (US, Switzerland and El Salvador) of the required two-thirds of WTO members have ratified the amendment. The requisite one hundred countries needed for formal ratification is unlikely to be reached given calls for a boycott on further ratification by LMICs and NGOs.

The legal status of the Doha Declaration and Paragraph 6 decision after 2007, therefore, remains uncertain, along with how its measures can be used to improve access to medicines. The report of the WHO Commission recommends a broad range of policy changes needed to improve all stages of drug production and use. For example, it calls on industrialised countries to allocate more resources to R&D on the health needs of LMICs, create ways of better sharing information, strengthen health delivery systems, and promote public-private partnerships. In relation to the Doha Declaration and Paragraph 6 decision specifically, the report calls for adaptations to national legislation and institutions to allow TRIPS flexibilities to be used, for public health justifications to be used when requiring data protection rules, and for the Paragraph 6 decision "to be kept under review and appropriate changes considered to achieve a workable solution, if necessary."[52]

While this paper supports the above recommendations, there is also a need to address the broader context of inequalities in power and influence within the global economy. The access to medicines issue mirrors the existing world trading system, formally governed by the WTO's 149 member states but, in practice, a product of stark inequalities within and across countries. Foremost is the need to recognise the powerful vested interests behind the globalization of IPRs, both government and corporate, often working collaboratively to further shared objectives. Drahos and Braithwaite describe TRIPS itself as the product of a corporate agenda, with its negotiation process highly skewed in favour of such interests [53]. While the Doha Declaration and Paragraph 6 decision were hard won agreements by public health advocates, Faunce asks to what extent the agreements are genuine commitments to improving access to medicines, or vaguely worded documents designed all along to give little away? [54]

Given the power and influence of the vested interests concerned, how can the public health community move forward to secure access to medicines? First, the current amendment to the TRIPS agreement should not be adopted. Instead, negotiations for simplified procedures under the Doha Declaration and Paragraph 6 decision, which enable the practical implementation of their measures, need to be initiated. The implications of the currently proposed amendment are still yet not fully understood, but the lack of compulsory licensing since 2003 is very concerning for LMICs. The action by the Thai government should be watched closely in this respect, along with consideration of how procedures impact LDCs with considerably less capacity and greater dependence on drug imports.

Second, LMICs with substantial pharmaceutical markets, such as India, Brazil and Thailand, can provide leadership and establish importance precedence by asserting the flexibilities available under TRIPS to protect public health. Countries with established generic manufacturing capacity, such as India and China, should protect access by adopting TRIPS flexibilities into national patent laws. For example, Chile took proactive steps to protect access to medicines from data exclusivity provisions even in the wake of signing its FTA with the US.

Third, and relatedly, LMICs and public health advocates can work collectively to resist pressures to dilute public health protections. In bilateral and regional trade negotiations, individual countries are especially vulnerable to the negotiating power of major trading nations. Joint efforts and combined forces are critical to the power imbalances inherent in trade negotiations. In May 2006, ten countries issued the Declaration of Ministers of South America over Intellectual Property, Access to Medicines, and Public Health. The declaration establishes a united position against the further spread of TRIPS-plus measures. The case of Rwanda suggests that even relatively small countries can cite public health need to negotiate better deals. As a recipient of PEPFAR funding, the country received US$ millions with the proviso that drugs approved by the US Federal Drug Administration (FDA), almost all brand named and manufactured by US-based companies, must be purchased. Recognising the higher cost of this proviso, the Rwandan government passed a law requiring the purchase of generic drugs when available for any and all treatment programs. The US yielded through an elaborate collaboration with other donors, and Rwanda was thus able to reduce the cost of drugs purchased and increase the number of patients treated [55]. Rwanda's ARV requirement was pooled, and the US became responsible for purchasing the brand name drugs required, while other funds went towards generic purchase. The drugs were then appropriately distributed to each site.

Fourth, "South-South" partnerships could be used to mitigate resource constraints, weaknesses in capacity and market failures. LMICs with established pharmaceutical industries could lead efforts in innovation and technology transfer [56]. An example is the Technological Network on AIDS, an initiative by Argentina, Brazil, China, Cuba, Nigeria, Russia, Ukraine and Thailand, to promote technology transfer [57]. Pooled procurement among LMICs, advocated by WHO, can also be effective for negotiating lower prices by combining markets and improving economies of scale. One example is the Organization of Eastern Caribbean States (OECS), representing nine Caribbean countries, which successfully reduced drug prices in the 1980s by about 44% compared to the original prices in the individual countries.

Fiftth, public health protections should be recognised as a starting point within all trade agreements, whether negotiated at the multilateral, regional and bilateral levels, and should be set aside from high-level "horse trading" that routinely takes place between negotiating parties. LMICs are not the only countries that would benefit from the protection of public health under TRIPS. The issue of access to medicines to meet critical public health needs arose during the deliberate spread of anthrax in the US by unknown parties in 2001. A potential shortage of the antibiotic Cipro^® ^(ciprofloxacin) prompted calls for the manufacturer Bayer to agree a voluntary license. After intense negotiations, the US and Canada reached agreement in October 2001 for Bayer to supply increased amounts of the drug at a "substantially lowered price" [58]. The agreement correctly took the chance that a major terrorist attack would not occur immediately and stockpiles could be built up over several years. Since 2003, similar concerns have been expressed over the need to stockpile anti-viral drugs in the event of an influenza pandemic. Once again a worldwide shortage of a patented medicine, in this case of oseltamivir phosphate (Tamiflu^®^), prompted debates about the importance of protecting IPRs versus protecting public health. While the use of compulsory licensing was avoided in both cases, they demonstrated that LMICs are not alone in their vulnerability to major public health threats. In the wake of the anthrax scare, the US government expressed its commitment to public health on a number of occasions, [59] including references to the Doha Declaration as one of four principal negotiating objectives for IPRs in the 2002 Trade Promotion Authorization Act. This act authorises the President to send signed trade agreements to Congress for consideration under expedited procedure [60]. In practice, the US trade representative has ignored the Doha Declaration in bilateral trade negotiations, instead leading the push for the globalization of more stringent IPRs [61-63]. The enhanced capacity of diseases to spread across borders as a result of globalization means that undermining the capacity of LMICs, in this way, may prove short sighted. While poor countries are clearly more vulnerable than others to public health threats, no country remains out of reach in a world of increasing globalization.

Finally, the role of the pharmaceutical industry is critical to this debate given its vital role in discovering and developing effective drugs. It remains among the Fortune 500's most profitable business sectors, [64,65] although it has not proven immune to setbacks. The expiration of patent rights on high profit products, intense competition from generics, failure to develop a new generation of "blockbuster" drugs, and public criticism have all cast shadows on the industry. The clear tensions between profit making and public interest are not easily resolved. The market alone will not resolve the problem of access to medicines by the world's poor. For pharmaceutical companies seeking world markets, the globalization of IPRs are seen as essential for recouping investment to develop and market new drugs, estimated (and disputed by some groups [66]) at US$802 million per drug [67]. Moreover, access to medicines is seen by many industry representatives as a problem arising from improper prescribing, irrational use and selection, poor distribution chains, and unsustainable financing [68].

The problem of access to medicines in LMICs is indeed linked to wider development needs, and undoubtedly may become less pressing as economic and social progress is achieved. However, as argued by the WHO Commission on Macroeconomics and Health, good health is an essential ingredient to poverty reduction and socioeconomic development [69]. Fighting disease is vital to economic success. The collective neglect of public health needs in LMICs maintains the vicious cycle of poor health and underdevelopment. Finding ways of improving access, correspondingly, can contribute to an upward spiral of better health and more rapid development. For pharmaceutical companies, the creation of new markets in LMICs may offer longer term sustainability and growth. It is estimated by the Global Alliance for TB Drug Development, for example, that the market for anti-TB drugs will grow, from around US$412–470 million in 2006 to US$612–670 million by 2010 [70].

## Conclusion

While the Doha Declaration and Paragraph 6 decision affirm important principles under the TRIPS agreement, regarding the protection of public health within international trade law, key challenges remain. The lack of progress in implementing TRIPS flexibilities to improve access to medicines, and the spread of TRIPS-plus measures through bilateral and regional trade agreements, require concerted attention. LMICs dependent on access to export markets in industrialised countries have been pressured to prioritise trade over public health protections. Powerful trading nations, acting on behalf of transnational pharmaceutical companies, have benefited from a "divide and conquer" strategy [71].

The challenge of improving access to medicines for LMICs thus stands at a critical crossroad. One choice is for the global community to allow the Doha Declaration to become a pawn in the high politics of trade policy, trampled by the spread of TRIPS-plus measures designed to push access to medicines by the poor even further out of reach. The other choice is to stand true to the public health protections available within the TRIPS agreement. This would mean an affirmation of those principles, setting them apart and above trade negotiations, accompanied by the commitment of sufficient resources to realise their potential.

## Abbreviations

ARV anti-retroviral

CAFTA Central American Free Trade Agreement

FTAA Free Trade Agreement of the Americas

HIV/AIDS human immunodeficiency syndrome/acquired immunodeficiency syndrome

IPR intellectual property rights

LDC least developed country

LMICs low and middle income countries

SACU Southern African Customs Union

TRIPS Agreement on Trade-Related Aspects of Intellectual Property Rights

WTO World Trade Organisation

## Competing interests

The author(s) declare that they have no competing interests.

## Authors' contributions

VBK conceptualised and drafted the paper and carried out an initial review of policy debates. KL revised the paper and provided additional review materials and analysis.
